# Frailty and pain in an internal medicine ward

**DOI:** 10.1590/1980-57642018dn13-010016

**Published:** 2019

**Authors:** Raquel Sousa Almeida, Maria João Pinto, João Matos Costa

**Affiliations:** 1 MD. Hospital Distrital de Santarém, Portugal - Department of Internal Medicine

Frailty is a term usually defined as a syndrome of physiological decline in late life, characterized by a state of increased vulnerability to stressors. It entails a high risk of adverse outcomes, such as functional deterioration, institutionalization, hospitalization and death. Pain is also likely to have a serious impact on older people who have^1^ limited ability to respond to additional stressors.^2^

The belief that ‘‘pain is just a part of getting older” is pervasive among patients and providers, and this notion might hinder effective treatment for the problem.^3^

With both conditions common among the elderly, particularly in hospitalized patients, the possibility of a correlation between frailty and pain has been raised. Elderly patients comprise a large proportion of admissions to Internal Medicine wards, therefore, both frailty and pain are frequently observed in this setting.

In this letter, we report a small study which aimed to examine the correlation between frailty and pain in elderly patients admitted to an Internal Medicine ward. Consecutive patients, aged 65 or older, admitted during a 1-month period were recruited for the purposes of the study. All patients provided their written informed consent before their inclusion.

We measured frailty using the Portuguese version of Tilburg Frailty Indicator (TFI)^4^ and pain with the Pain Impact Questionnaire (PIQ-6).^5^ Patients less than 65 years of age or with severe intellectual impairment according to the Portable Mental Status Questionnaire,^6^ as well as individuals who refused to participate, were excluded.

Of the 63 elderly patients admitted, 41 were included in the study. The mean age of participants was 79±6.0 years and 70.7% of the patients were women. A total of 58.5% patients were considered frail (TFI score ≥6). Mean TFI total score was 6.0±2.6 points. Mean physical domain score was 3.5±1.6, psychological domain score 2.0±1.2 and social domain score 0.4±0.5 points.

Pain was reported in 80.5% of the patients, who had a mean score of 58.5±8.1 on the PIQ-6. There was a previous ambulatory prescription of pain medication in 9.8%. During admission, 56.1% had analgesic therapy (87.0% with paracetamol, as needed). At discharge, 11.1% had an analgesic therapy prescription.

Pain and frailty were higher in women and in the group aged >80 years, but this was not statistically significant (p=0.07).

**Figure 1:**
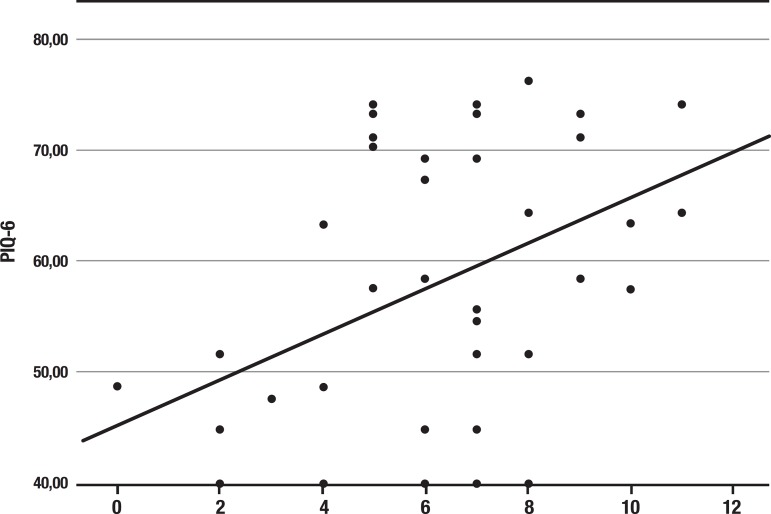
Correlation between Tilburg Frailty Indicator (TFI) score and Pain Impact Questionnaire (PIQ-6).

A higher PIQ-6 score was correlated with a higher total TFI, reaching statistical significance (r=0.442; p=0.004 - Figure 1).

In conclusion, higher levels of pain were correlated with higher levels of frailty. The concept of pain homeostenosis, where pain could diminish the physiological reserves needed to maintain homeostasis when faced with biological, psychological or social stressors, and therefore precipitate frailty, seems to be a well-supported hypothesis for this association.^1^^,^ 7 ^,^ 8

There are some limitations of this study. We recognize the multidimensional nature of frailty and pain, and that the lack of adjustment for depressive symptoms could be a bias in the study. Despite the small sample, our results emphasize the importance of early detection and interdisciplinary intervention for pain, especially in the elderly population, to help prevent vulnerability and reduce the incidence of complications. Hospitalization is an opportunity to intervene, but there is still much to be done in raising awareness of health professionals.
